# Possible tattoo-transmitted monkeypox viral infection

**DOI:** 10.1007/s11739-022-03090-x

**Published:** 2022-09-16

**Authors:** Carlo Tascini, Monica Geminiani, Francesco Sbrana, Alberto Pagotto, Luca Martini

**Affiliations:** 1 Department of Medicine (DAME), University of Udine, 33100 Udine, Italy; 2 Fondazione Toscana “Gabriele Monasterio”, Pisa, Italy; 3 U.O. Malattie Infettive, Azienda Sanitaria Universitaria Integrata di Udine, Via Pozzuolo, 330, 33100 Udine, Italy

Dear Editor,

A 39-year-old man referred to the Infectious Diseases Clinic of the Udine University Hospital (Italy) for lesions compatible with monkeypox disease. He reported fever and maculo-vesicular lesions typically associated with monkeypox within the previous 7 days. The patient’s referred having unprotected oral sex and protected sexual intercourse with 2 female partners in the previous 30 days. He also reported getting tattooed in Spain 7 days prior onset of symptoms.

**Fig. 1:**
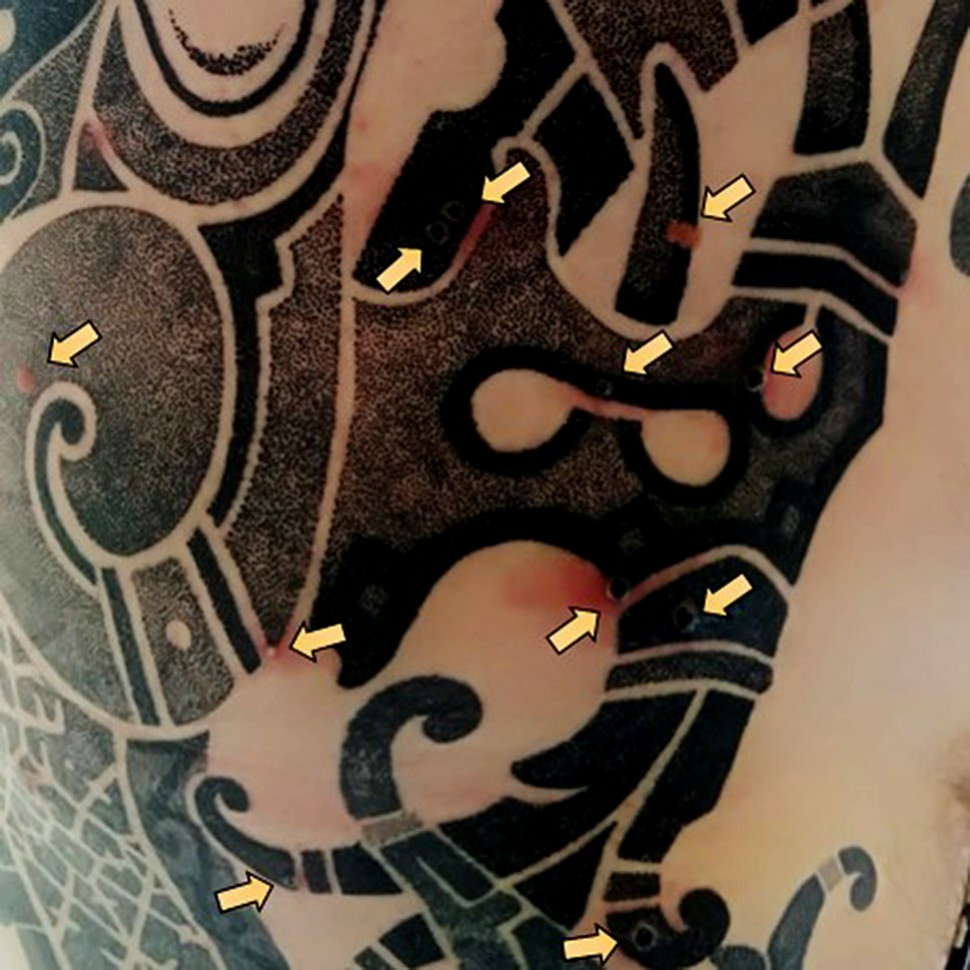
Tattoo skin area with monkeypox lesions (arrows)

Monkeypox lesions initially manifested within the tattooed skin area (Fig. 1). Swab testing of the area confirmed the presence of monkeypox by PCR assay. Notably, the patient was not vaccinated against smallpox. He was followed up at home without therapy. At follow-up visit after 7 days, his condition resolved completely.

Monkeypox transmission was ascribed to sexual contact in 95% of reported cases [1], although sexual transmission was never confirmed. Close contacts or use of fomites might indeed be the underlying cause of viral transmission. Generally, monkeypox infection was associated with factors such as multiple sexual partners within the previous 3 months, travel abroad in the previous month and participation to pride events or visit to sex-on-site venues in the previous month in approximately 20% of reported cases [2]. With the exception of the latter, the hereby reported case presented with all other risk factors. Curiously, however, in this instance, the first lesions appeared on the site of tattoo, performed 7 days before the appearance of symptoms. While close sexual contact could not be out ruled, we nonetheless postulated that the tattoo might be the source of monkeypox infection. Indeed, a breakout of 12 cases related to tattoo centers in Spain was just recently reported [3].

Tattoos may not surprisingly be the source of contamination as indeed other cases of systemic viral disease transmission have been reported in the past (HIV, hepatitis B and C) as well as local viral infections such as Molluscum contagiosum, HPV and HSV1 and HSV2. In the latter 2 cases, incubation time is quite brief accounting for only approximately 3 days [4]. Moreover, in local viral infections, lesions typically appear in the tattoo area.

To conclude, our report calls for renewed attention in activities involving close contacts such as tattoo shops, whereby particular attention should be placed on guaranteeing sterilization of tools and ink to minimize transmission of infective agents.
